# 1-(2,4-Dichloro­benzyl­idene)-4-ethyl­thio­semicarbazide

**DOI:** 10.1107/S1600536810035671

**Published:** 2010-09-11

**Authors:** Yu-Feng Li

**Affiliations:** a Microscale Science Institute, Department of Chemistry and Chemical Engineering, Weifang University, Weifang 261061, People’s Republic of China

## Abstract

The title compound, C_10_H_11_Cl_2_N_3_S, was prepared by the reaction of 4-ethyl­thio­semicarbazide and 2,4-dichloro­benzaldehyde. It is approximately planar, the dihedral angle between the benzene ring and the thio­urea unit being 8.43 (18)°. In the crystal, inversion dimers linked by pairs of N—H⋯S hydrogen bonds generate *R*_2_^2^(8) loops.

## Related literature

For background to Schiff bases, see: Casas *et al.* (2000 ▶). For a related structure, see: Li & Jian (2010 ▶).
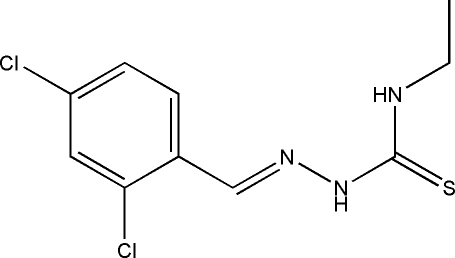


## Experimental

### 

#### Crystal data

C_10_H_11_Cl_2_N_3_S
*M* *_r_* = 276.18
Monoclinic, 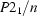

*a* = 5.4339 (11) Å
*b* = 20.526 (4) Å
*c* = 11.313 (2) Å
β = 101.97 (3)°
*V* = 1234.4 (4) Å^3^
*Z* = 4
Mo *K*α radiation
μ = 0.67 mm^−1^
*T* = 293 K
0.22 × 0.20 × 0.18 mm

#### Data collection

Bruker SMART CCD diffractometer
10913 measured reflections
2707 independent reflections
1416 reflections with *I* > 2σ(*I*)
*R*_int_ = 0.109

#### Refinement

*R*[*F* ^2^ > 2σ(*F* ^2^)] = 0.060
*wR*(*F* ^2^) = 0.187
*S* = 0.92
2707 reflections
145 parameters
H-atom parameters constrained
Δρ_max_ = 0.37 e Å^−3^
Δρ_min_ = −0.35 e Å^−3^

### 

Data collection: *SMART* (Bruker, 1997 ▶); cell refinement: *SAINT* (Bruker, 1997 ▶); data reduction: *SAINT*; program(s) used to solve structure: *SHELXS97* (Sheldrick, 2008 ▶); program(s) used to refine structure: *SHELXL97* (Sheldrick, 2008 ▶); molecular graphics: *SHELXTL* (Sheldrick, 2008 ▶); software used to prepare material for publication: *SHELXTL*.

## Supplementary Material

Crystal structure: contains datablocks global, I. DOI: 10.1107/S1600536810035671/hb5632sup1.cif

Structure factors: contains datablocks I. DOI: 10.1107/S1600536810035671/hb5632Isup2.hkl

Additional supplementary materials:  crystallographic information; 3D view; checkCIF report

## Figures and Tables

**Table 1:** Hydrogen-bond geometry (Å, °)

*D*—H⋯*A*	*D*—H	H⋯*A*	*D*⋯*A*	*D*—H⋯*A*
N2—H2*A*⋯S1^i^	0.86	2.56	3.409 (5)	168

Symmetry code: (i) 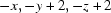.

**Fig. 1.**
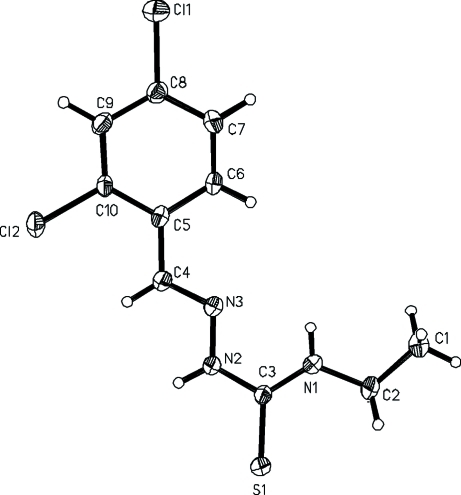
The structure of (I) showing 30% probability displacement ellipsoids.

## supplementary crystallographic information

### Comment

Schiff-base have attracted much attention because they can be utilized as effective ligands to be coordination compounds in coordination chemistry. (Casas *et al.*, 2000). As part of our research for new Schiff-base compounds we synthesized the title compound (I), and describe its structure here. In the molecule structure, the dihedral angle between the benzene ring and the thiourea unit is [8.43 (18)°].

Bond lengths and angles agree with those observed in a related structure (Li & Jian, 2010).

### Experimental

A mixture of 4-ethylthiosemicarbazide (0.1 mol) and 2,4-dichlorobenzaldehyde (0.1 mol) was stirred in refluxing ethanol (30 mL) for 2 h to afford the title compound (0.090 mol, yield 90%). Colourless blocks of (I) were obtained by recrystallization from ethanol at room temperature.

### Refinement

H atoms were fixed geometrically and allowed to ride on their attached atoms, with C—H distances=0.97 Å, and with *U*_iso_=1.2–1.5*U*_eq_.

### Crystal data

**Table:**

C_10_H_11_Cl_2_N_3_S	*F*(000) = 568
*M**_r_* = 276.18	*D*_x_ = 1.486 Mg m^−^^3^
Monoclinic, *P*2_1_/*n*	Mo *K*α radiation, λ = 0.71073 Å
Hall symbol: -P 2yn	Cell parameters from 1416 reflections
*a* = 5.4339 (11) Å	θ = 3.5–27.5°
*b* = 20.526 (4) Å	µ = 0.67 mm^−^^1^
*c* = 11.313 (2) Å	*T* = 293 K
β = 101.97 (3)°	Block, colorless
*V* = 1234.4 (4) Å^3^	0.22 × 0.20 × 0.18 mm
*Z* = 4	

### Data collection

**Table:**

Bruker SMART CCD diffractometer	1416 reflections with *I* > 2σ(*I*)
Radiation source: fine-focus sealed tube	*R*_int_ = 0.109
graphite	θ_max_ = 27.5°, θ_min_ = 3.5°
phi and ω scans	*h* = −6→6
10913 measured reflections	*k* = −26→26
2707 independent reflections	*l* = −14→14

### Refinement

**Table:**

Refinement on *F*^2^	Primary atom site location: structure-invariant direct methods
Least-squares matrix: full	Secondary atom site location: difference Fourier map
*R*[*F*^2^ > 2σ(*F*^2^)] = 0.060	Hydrogen site location: inferred from neighbouring sites
*wR*(*F*^2^) = 0.187	H-atom parameters constrained
*S* = 0.92	*w* = 1/[σ^2^(*F*_o_^2^) + (0.1*P*)^2^] where *P* = (*F*_o_^2^ + 2*F*_c_^2^)/3
2707 reflections	(Δ/σ)_max_ < 0.001
145 parameters	Δρ_max_ = 0.37 e Å^−^^3^
0 restraints	Δρ_min_ = −0.35 e Å^−^^3^

### Special details

**Table:**

Geometry. All e.s.d.'s (except the e.s.d. in the dihedral angle between two l.s. planes) are estimated using the full covariance matrix. The cell e.s.d.'s are taken into account individually in the estimation of e.s.d.'s in distances, angles and torsion angles; correlations between e.s.d.'s in cell parameters are only used when they are defined by crystal symmetry. An approximate (isotropic) treatment of cell e.s.d.'s is used for estimating e.s.d.'s involving l.s. planes.
Refinement. Refinement of *F*^2^ against ALL reflections. The weighted *R*-factor *wR* and goodness of fit *S* are based on *F*^2^, conventional *R*-factors *R* are based on *F*, with *F* set to zero for negative *F*^2^. The threshold expression of *F*^2^ > σ(*F*^2^) is used only for calculating *R*-factors(gt) *etc*. and is not relevant to the choice of reflections for refinement. *R*-factors based on *F*^2^ are statistically about twice as large as those based on *F*, and *R*- factors based on ALL data will be even larger.

### Fractional atomic coordinates and isotropic or equivalent isotropic displacement parameters (Å^2^)

**Table:**

	*x*	*y*	*z*	*U*_iso_*/*U*_eq_	
S1	−0.1063 (2)	0.99987 (6)	0.79802 (13)	0.0434 (4)	
Cl2	0.7303 (2)	0.85591 (6)	1.34950 (13)	0.0499 (4)	
Cl1	1.4115 (2)	0.70212 (6)	1.20880 (15)	0.0558 (4)	
N3	0.4756 (6)	0.90330 (15)	0.9707 (4)	0.0355 (9)	
N2	0.2667 (7)	0.94274 (17)	0.9454 (4)	0.0390 (9)	
H2A	0.2030	0.9583	1.0029	0.047*	
N1	0.2795 (7)	0.93533 (18)	0.7450 (4)	0.0410 (10)	
H1A	0.4117	0.9118	0.7681	0.049*	
C5	0.7795 (7)	0.84768 (18)	1.1156 (4)	0.0317 (10)	
C3	0.1623 (7)	0.95672 (19)	0.8288 (4)	0.0324 (10)	
C8	1.1713 (8)	0.7596 (2)	1.1742 (5)	0.0381 (11)	
C9	1.0643 (8)	0.78286 (19)	1.2644 (5)	0.0393 (12)	
H9A	1.1209	0.7695	1.3440	0.047*	
C4	0.5640 (8)	0.89200 (19)	1.0829 (5)	0.0373 (11)	
H4A	0.4932	0.9114	1.1423	0.045*	
C7	1.0936 (9)	0.7793 (2)	1.0562 (5)	0.0418 (12)	
H7A	1.1715	0.7632	0.9964	0.050*	
C10	0.8684 (8)	0.82717 (19)	1.2342 (4)	0.0332 (10)	
C6	0.8994 (8)	0.8232 (2)	1.0276 (5)	0.0384 (11)	
H6A	0.8471	0.8368	0.9480	0.046*	
C2	0.2011 (10)	0.9487 (3)	0.6169 (5)	0.0492 (13)	
H2B	0.2201	0.9949	0.6029	0.059*	
H2C	0.0246	0.9377	0.5906	0.059*	
C1	0.3510 (11)	0.9109 (3)	0.5440 (6)	0.0641 (16)	
H1B	0.2935	0.9209	0.4599	0.096*	
H1C	0.3306	0.8651	0.5567	0.096*	
H1D	0.5255	0.9223	0.5686	0.096*	

### Atomic displacement parameters (Å^2^)

**Table:**

	*U*^11^	*U*^22^	*U*^33^	*U*^12^	*U*^13^	*U*^23^
S1	0.0362 (7)	0.0577 (7)	0.0350 (8)	0.0114 (4)	0.0044 (5)	0.0005 (5)
Cl2	0.0628 (8)	0.0614 (7)	0.0280 (8)	0.0123 (5)	0.0151 (6)	0.0009 (6)
Cl1	0.0561 (8)	0.0551 (7)	0.0546 (11)	0.0202 (5)	0.0078 (7)	0.0014 (6)
N3	0.037 (2)	0.0394 (19)	0.030 (3)	0.0028 (13)	0.0062 (17)	0.0035 (16)
N2	0.039 (2)	0.051 (2)	0.026 (3)	0.0122 (15)	0.0047 (17)	0.0013 (17)
N1	0.037 (2)	0.054 (2)	0.030 (3)	0.0091 (15)	0.0019 (18)	0.0029 (18)
C5	0.039 (2)	0.030 (2)	0.025 (3)	−0.0023 (15)	0.0038 (19)	0.0018 (17)
C3	0.031 (2)	0.040 (2)	0.026 (3)	−0.0020 (16)	0.0043 (19)	−0.0015 (18)
C8	0.040 (2)	0.038 (2)	0.034 (3)	0.0015 (17)	0.003 (2)	−0.0022 (19)
C9	0.045 (3)	0.041 (2)	0.029 (3)	0.0050 (17)	−0.001 (2)	0.0030 (19)
C4	0.038 (2)	0.042 (2)	0.030 (3)	0.0049 (17)	0.004 (2)	−0.0011 (19)
C7	0.045 (3)	0.047 (2)	0.035 (3)	0.0042 (18)	0.012 (2)	−0.007 (2)
C10	0.041 (2)	0.037 (2)	0.022 (3)	0.0003 (16)	0.0089 (19)	−0.0005 (18)
C6	0.045 (3)	0.047 (2)	0.023 (3)	0.0012 (18)	0.005 (2)	−0.003 (2)
C2	0.055 (3)	0.066 (3)	0.025 (3)	0.011 (2)	0.004 (2)	0.004 (2)
C1	0.057 (3)	0.099 (4)	0.038 (4)	0.015 (3)	0.013 (3)	−0.004 (3)

### Geometric parameters (Å, °)

**Table:**

S1—C3	1.681 (4)	C8—C7	1.376 (7)
Cl2—C10	1.738 (5)	C9—C10	1.388 (6)
Cl1—C8	1.743 (4)	C9—H9A	0.9300
N3—C4	1.282 (6)	C4—H4A	0.9300
N3—N2	1.375 (5)	C7—C6	1.373 (6)
N2—C3	1.354 (6)	C7—H7A	0.9300
N2—H2A	0.8600	C6—H6A	0.9300
N1—C3	1.322 (6)	C2—C1	1.491 (7)
N1—C2	1.449 (7)	C2—H2B	0.9700
N1—H1A	0.8600	C2—H2C	0.9700
C5—C6	1.392 (7)	C1—H1B	0.9600
C5—C10	1.393 (6)	C1—H1C	0.9600
C5—C4	1.468 (6)	C1—H1D	0.9600
C8—C9	1.362 (7)		
			
C4—N3—N2	115.8 (4)	C6—C7—C8	119.2 (5)
C3—N2—N3	119.2 (4)	C6—C7—H7A	120.4
C3—N2—H2A	120.4	C8—C7—H7A	120.4
N3—N2—H2A	120.4	C9—C10—C5	122.1 (4)
C3—N1—C2	124.7 (4)	C9—C10—Cl2	117.8 (4)
C3—N1—H1A	117.7	C5—C10—Cl2	120.2 (3)
C2—N1—H1A	117.7	C7—C6—C5	121.3 (5)
C6—C5—C10	117.3 (4)	C7—C6—H6A	119.3
C6—C5—C4	120.7 (4)	C5—C6—H6A	119.3
C10—C5—C4	121.9 (4)	N1—C2—C1	111.9 (4)
N1—C3—N2	117.5 (4)	N1—C2—H2B	109.2
N1—C3—S1	123.6 (4)	C1—C2—H2B	109.2
N2—C3—S1	118.9 (4)	N1—C2—H2C	109.2
C9—C8—C7	122.1 (4)	C1—C2—H2C	109.2
C9—C8—Cl1	119.0 (4)	H2B—C2—H2C	107.9
C7—C8—Cl1	118.8 (4)	C2—C1—H1B	109.5
C8—C9—C10	118.0 (5)	C2—C1—H1C	109.5
C8—C9—H9A	121.0	H1B—C1—H1C	109.5
C10—C9—H9A	121.0	C2—C1—H1D	109.5
N3—C4—C5	118.5 (5)	H1B—C1—H1D	109.5
N3—C4—H4A	120.8	H1C—C1—H1D	109.5
C5—C4—H4A	120.8		

### Hydrogen-bond geometry (Å, °)

**Table:**

*D*—H···*A*	*D*—H	H···*A*	*D*···*A*	*D*—H···*A*
N2—H2A···S1^i^	0.86	2.56	3.409 (5)	168

Symmetry codes: (i) −*x*, −*y*+2, −*z*+2.

## References

[bb1] Bruker (1997). *SMART* and *SAINT* Bruker AXS Inc., Madison, Wisconsin, USA.

[bb2] Casas, J. S., Garcia-T, M. S. & Sordo, J. (2000). *Coord. Chem. Rev.* **209**, 197–261.

[bb3] Li, Y.-F. & Jian, F.-F. (2010). *Acta Cryst.* E**66**, o1399. 10.1107/S1600536810017988 PMC2979434 21579478

[bb4] Sheldrick, G. M. (2008). *Acta Cryst.* A**64**, 112–122. 10.1107/S0108767307043930 18156677

